# Left atrial dysfunction detected by speckle tracking in patients with systemic sclerosis

**DOI:** 10.1186/1476-7120-12-30

**Published:** 2014-08-05

**Authors:** Gergely Agoston, Luna Gargani, Marcelo Haertel Miglioranza, Maria Caputo, Luigi Paolo Badano, Antonella Moreo, Denisa Muraru, Sergio Mondillo, Alberto Moggi Pignone, Marco Matucci Cerinic, Rosa Sicari, Eugenio Picano, Albert Varga

**Affiliations:** 1 2nd Department of Medicine and Cardiology Center, University of Szeged, Korányi Fasor 6, 6720 Szeged, Hungary; 2 Institute of Clinical Physiology, National Council of Research, Pisa, Italy; 3 Department of Cardiac, Thoracic and Vascular Sciences, University of Padua, Padua, Italy; 4 Cardiology Institute of Rio Grande do Sul, Porto Alegre, Brazil; 5 Department of Cardiovascular Diseases, University of Siena, Siena, Italy; 6 Cardiology Department, Niguarda Ca’ Granda Hospital, Milan, Italy; 7 Department of Internal Medicine, University of Florence, Florence, Italy; 8 Department of Biomedicine, Division of Rheumatology, University of Florence, Florence, Italy

## Abstract

**Background:**

Cardiac involvement is a relevant clinical finding in systemic sclerosis (SSc) and is associated with poor prognosis. Left atrial (LA) remodeling and/or dysfunction can be an early sign of diastolic dysfunction. Two-dimensional speckle tracking echocardiography (STE) is a novel and promising tool for detecting very early changes in LA myocardial performance.

**Aim:**

To assess whether STE strain parameters may detect early alterations in LA function in SSc patients.

**Methods:**

Forty-two SSc patients (Group 1, age 50 ± 14 years, 95% females) without clinical evidence for cardiac involvement and 42 age- and gender-matched control subjects (Group 2, age 49 ± 13 years, 95% females) were evaluated with comprehensive 2D and Doppler echocardiography, including tissue Doppler imaging analysis. Positive peak left atrial longitudinal strain (ϵ _pos peak_), second positive left atrial longitudinal strain (sec ϵ _pos peak_), and negative left atrial longitudinal strain (ϵ _neg peak_) were measured using a 12-segment model for the LA, by commercially available semi-automated 2D speckle-tracking software (EchoPac PC version 108.1.4, GE Healthcare, Horten, Norway).

**Results:**

All SSc patients had a normal left ventricular ejection fraction (63.1 ± 4%). SSc patients did not differ from controls in E/A (Group 1 = 1.1 ± 0.4 vs Group 2 = 1.3 ± 0.4, p = .14) or pulmonary arterial systolic pressure (Group 1 = 24.1 ± 8 mmHg vs Group 2 = 21 ± 7 mmHg, p = .17). SSc patients did not show significantly different indexed LA volumes (Group 1 = 24.9 ± 5.3 ml/m^2^ vs Group 2 = 24.7 ± 4.4 ml/m^2^, p = .8), whereas E/e’ ratio was significantly higher in SSc (Group 1 = 7.6 ± 2.4 vs Group 2 = 6.5 ± 1.7, p<0.05), although still within normal values. LA strain values were significantly different between the two groups (ϵ _pos peak_ Group 1 = 31.3 ± 4.2% vs Group 2 = 35.0 ± 7.6%, p < .01, sec ϵ _pos peak_ Group 1 = 18.4 ± 4 vs Group 2 = 21.4 ± 7.6, p < 0.05).

**Conclusion:**

2D speckle-tracking echocardiography is a sensitive tool to assess impairment of LA mechanics, which is detectable in absence of changes in LA size and volume, and may represent an early sign of cardiac involvement in patients with SSc.

## Introduction

Systemic sclerosis (SSc) is a chronic, systemic connective tissue disease characterized by inflammation and fibrosis involving various organs, including the skin, lungs, gastrointestinal tract, kidneys and heart. Although cardiac involvement is often clinically asymptomatic [1], it occurs in a significant percentage of patients [2,3]. All cardiac structures - endocardium, myocardium, pericardium, valves, coronary arteries, electrical and nervous system - may be involved, potentially leading to heart failure. Primary myocardial involvement, without systemic or pulmonary hypertension and without significant renal or pulmonary involvement, implicates different pathophysiological mechanisms, including the characteristic vascular lesions and fibrosis deposition, which may impair coronary microcirculation and myocardial function, and is one of the leading causes of mortality in these patients [4]. Systolic and/or diastolic dysfunction can develop very early in the course of the disease, even years before becoming clinically relevant, and are recognized as very powerful adverse prognostic factors [5]. Preclinical identification of cardiac involvement is pivotal for adequate early management of these patients.

Left atrial (LA) volume, as an LA functional index, has recently been identified as a potential marker of cardiac involvement and atrial arrhythmias [6]. Changes in LA volumes and mechanics have been demonstrated in patients with rheumatic disease, and can even precede the involvement of the ventricles [7,8]. Recently, the assessment of LA deformation profiles obtained by deformation imaging has been proposed as an alternative method of exploring LA function and to detect early changes in LA myocardial performance [9,10]. Our aim was to evaluate LA function in SSc patients by two-dimensional speckle tracking echocardiography (STE) strain parameters.

## Methods

### Study population

From September 2009 to January 2010, 42 consecutive patients affected with SSc (Group 1, age 50 ± 14 years, 95% females) admitted to the Rheumatology Clinic of Florence, and 42 age and gender-matched control subjects (Group 2, age 49 ± 13 years, and 95% females) were enrolled. Patients in Group 1 underwent a thorough clinical characterization, including a modified Rodnan skin score [11], pulmonary function test [12], assessment of pulmonary fibrosis by standard chest X-ray, lung ultrasound [13] and, when clinically indicated, by thoracic high-resolution computed tomography scan [14]. Inclusion criteria were: 1) age > 18 and < 85 years; 2) a previous diagnosis of SSc according to the American Rheumatism Association classification criteria for SSc [15]. Exclusion criteria were: 1) inability to provide informed consent; 2) known history of coronary artery disease, electrocardiographic signs of myocardial ischemia, left ventricular ejection fraction <55%, regional wall motion abnormalities, left ventricular hypertrophy, more than mild valvular heart disease, pericardial effusion, and evidence or clear history of atrial fibrillation, inadequate LA tracking for strain analysis. Anticentromere antibodies (ACA by indirect immunofluorescence on Hep-2 cells and by ELISA for CENP antigen) and antitopoisomerase I antibodies (anti-Scl70 by immunoblot analysis) were determined. All operators were unaware of the results of the other tests. The local Ethical Committee of Pisa, Italy, protocol number 2849 approved and all patients gave informed consent.

### Echocardiography

All patients underwent comprehensive two-dimensional transthoracic echocardiography examinations at rest, using conventional methods with a commercially available ultrasound machine (Vivid 7, GE Medical Systems, Horten, Norway) equipped with a 2.5-3.5 MHz phased-array sector scan probe, second harmonic technology, and coupled with tissue Doppler imaging (TDI). Left ventricular (LV) end-diastolic and end-systolic diameters were measured from the internal dimensions obtained from parasternal long axis view. LA diameters were measured from the apical four-chamber view. LA areas and volumes were measured using the biplane method of disks (modified Simpson’s rule), in the apical 4- and 2-chamber view at end-systole (maximum LA size), and a mean value of area and volume was obtained [16]. LA volumes were subsequently indexed to body surface area (BSA). LV mass was calculated by the Devereux formula and then indexed to body surface area [16]. Mitral regurgitation was assessed semi-quantitatively (0 = absent or trivial, 1 = mild, 2 = moderate, 3 = severe), including evaluation of vena contracta, regurgitant volume and effective regurgitant orifice area, when indicated [17]. TDI was evaluated, as previously described, in the pulsed-wave Doppler mode, to assess longitudinal myocardial regional LV function. A volume was sampled centrally to the basal segment of infero-septal and antero-lateral wall for the LV, and then the mean value of the velocity profiles was recorded. Gain and filters were adjusted as needed, to eliminate background noise and to obtain a clear tissue signal. TDI signals were recorded at a sweep of 100 mm/s. Each parameter was measured as the average of at least three consecutive beats. LV diastolic function was determined from the pattern of mitral flow velocity by pulsed Doppler echocardiography, complemented by mitral annular velocity by TDI and LA volumes. Diastolic dysfunction was graded as “absent” (grade 0), “mild” (grade 1, impaired relaxation), “moderate” (grade 2, pseudonormalized filling pattern), and “severe” (grade 3, restrictive filling pattern) [18]. Pulmonary artery systolic pressure (PASP) was estimated from peak tricuspid regurgitation jet velocities, adding right atrial pressure estimated from inferior vena cava diameter and respiratory changes [19]. All measurements were performed according to the recommendations of the European Association of Echocardiography/American Society of Echocardiography [16-20].

### Assessment of the left atrial function

**Figure 1:**
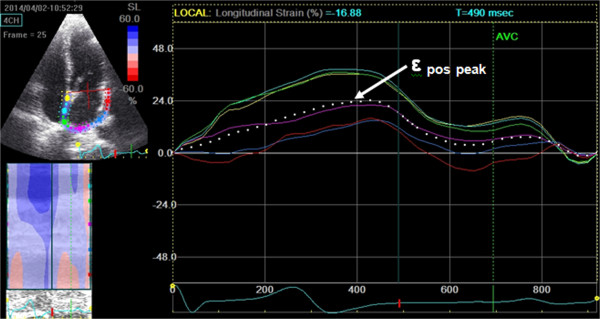
**2D speckle tracking derived LA peak, positive longitudinal strain (ϵ **_**pos peak**_**)measurement form apical 4 chamber view (the reference point is the beginning of the QRS complex).**

**Figure 2:**
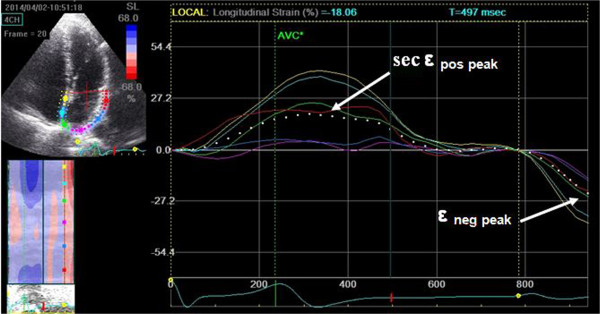
**Measurement of second positive peak longitudinal LA strain (sec ϵ**_**pos peak**_**) and negative peak longitudinal strain (ϵ**_**neg peak**_**) from apical four chamber view (the reference point is the onset of the p wave).**

Particular attention was paid to obtaining an adequate grayscale image, allowing reliable delineation of myocardial tissue and extracardiac structures. During breath hold, 3 consecutive heart cycles were recorded and averaged. The frame rate was set between 60 and 80 frames per second. These settings are recommended to combine temporal resolution with adequate spatial definition, and to enhance the feasibility of the frame-to frame tracking technique [21]. Recordings were processed using acoustic-tracking software (EchoPac PC version 108.1.4, GE Healthcare, Horten, Norway), allowing off-line semi-automated analysis of STE strain. In the end-diastolic/systolic frame, the atrial endocardial border was traced by a point-and-click method. After automatic creation of a region of interest, the LA wall was divided into six subregions, and segmental tracking quality was analyzed. We analyzed LA from apical two- and four-chamber views, so we used a 12-segment model. The dashed curve represents the average strain (Figure 1). The tracking settings allow distinguishing three LA strain values. If the reference point is set at the onset of the QRS, we can measure positive peak atrial longitudinal strain (ϵ _pos peak_), which corresponds to LA reservoir function (Figure 1). If the reference point is set at the onset of the P wave, we can measure both negative atrial longitudinal strain (ϵ _neg peak_), which mirrors LA pump function and second positive peak atrial strain (sec ϵ _pos peak_), which corresponds to LA conduit function [21] (Figure 2). Inter- and intra-observer variability of strain parameters has been previously assessed [9].

### Statistical analysis

Data are presented as mean ± standard deviation (SD) unless otherwise stated. Comparisons between SSc patients and controls were performed using Student’s t-tests or by non-parametric Mann–Whitney *U*-test, as appropriate. Comparisons between categorical variables were made with χ^2^ test. All tests were two-sided and p-values < .05 were considered statistically significant. Correlations were tested by Pearson or Spearman’s correlation tests, as appropriate. All analyses were performed using SPSS 20.0.0 (IBM Inc, Chicago, IL, USA) and GraphPad Prism version 6 (GraphPad Software Inc., San Diego, CA, USA).

## Results

**Figure 3:**
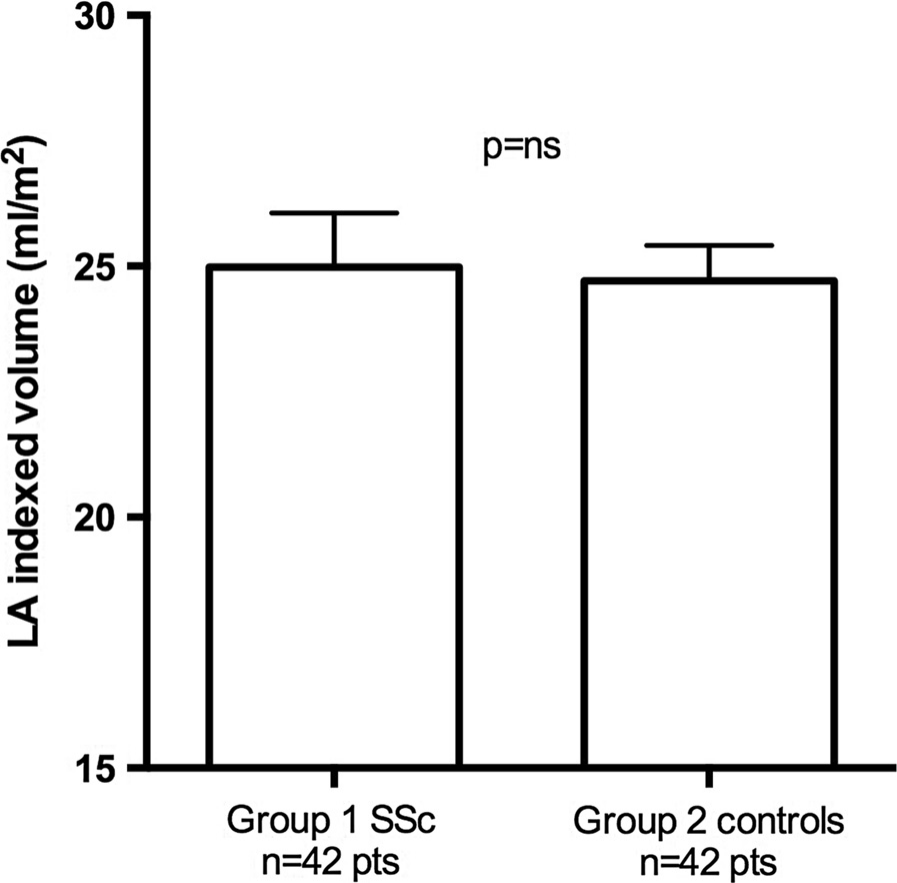
Differences in the indexed left atrial volume between SSc and control patients.

**Figure 4:**
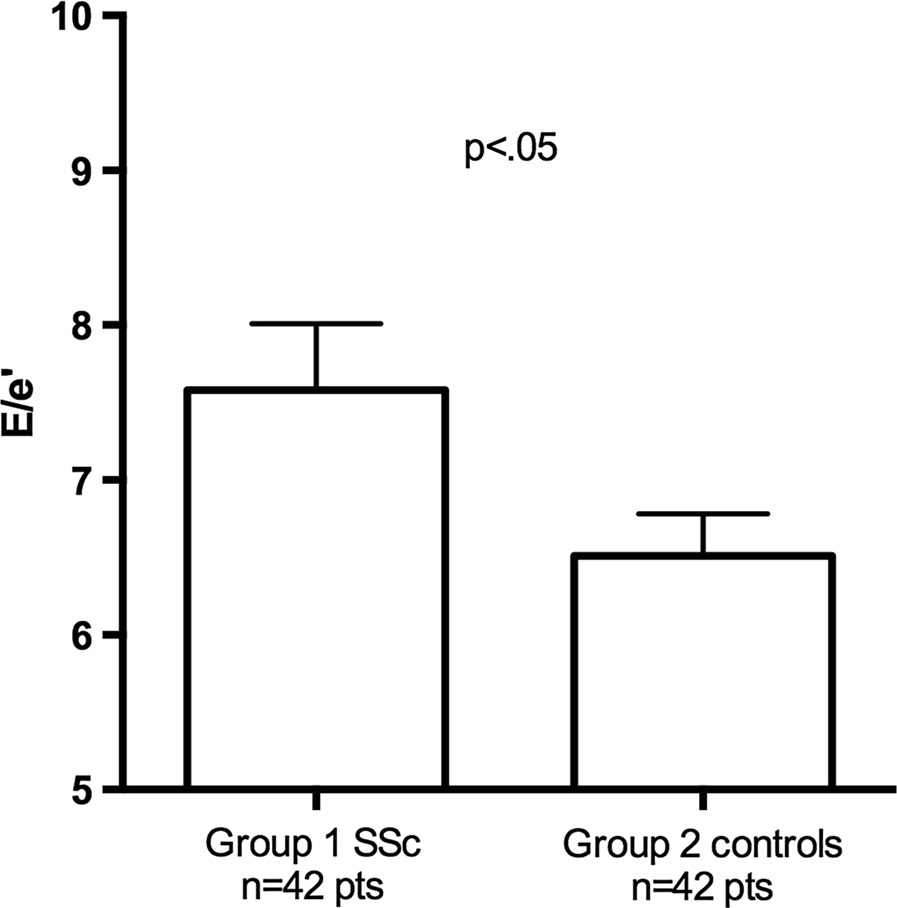
Differences in E/e’ between SSc and control patients.

**Figure 5:**
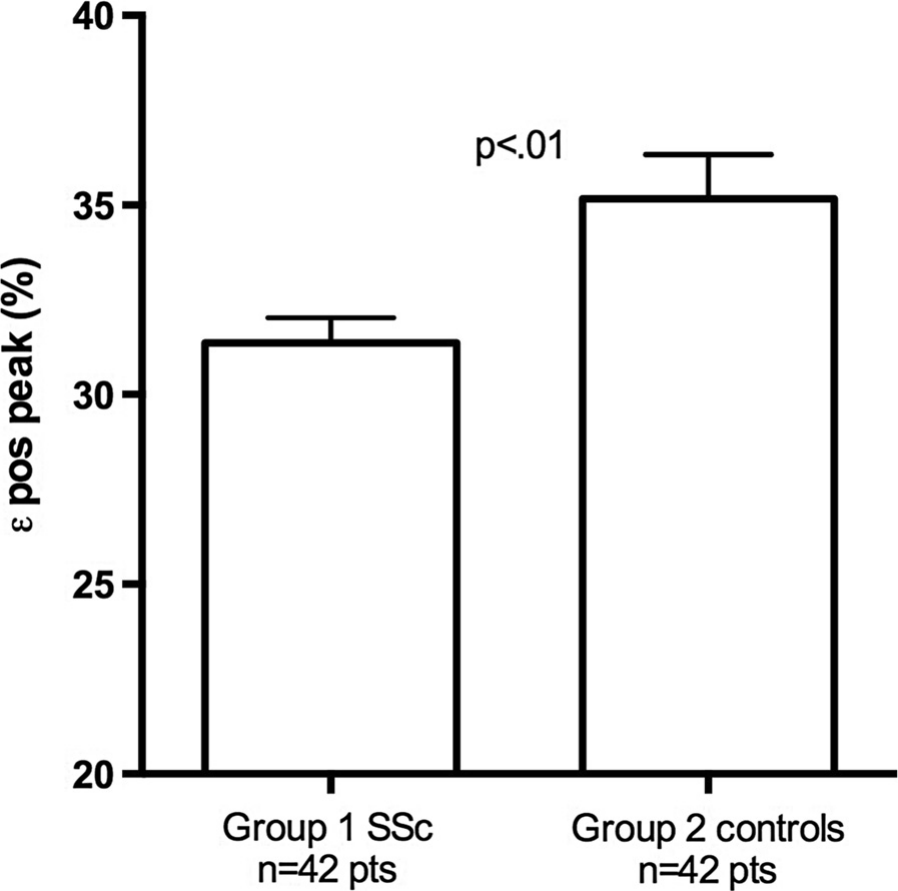
**Differences in peak positive LA longitudinal strain (ϵ **_**pos peak**_**) between SSc and control patients.**

The clinical and echocardiographic characteristics of Groups 1 and 2 are summarized in Tables 1 and 2. From the initial population of 55 patients, 7 were excluded for inadequate LA tracking quality, and 6 were excluded for the evidence of cardiac abnormalities (2 patients for EF < 55%, 3 patients for pericardial effusion, 1 patient for LV hypertrophy). Two patients in Group 1 had more than 1 cardiovascular risk factors (1 with arterial hypertension and smoking habit, and 1 with arterial hypertension and diabetes mellitus); Group 2 included 3 patients with concomitant arterial hypertension and dyslipidemia. The two groups did not differ in systolic function of the left ventricle (Group 1 EF = 63.1 ± 4%, vs Group 2 = 66.1 ± 4%, p = ns). SSc patients did not show significantly different LA indexed volumes (Group 1 = 24.9 ± 5.3 ml/m^2^ vs Group 2 = 24.7 ± 4.4 ml/m^2^, p = .8, Figure 3), but showed significantly different LV diastolic and LA longitudinal strain parameters. E/e’ ratio was higher in SSc patients (Group 1 = 7.6 ± 2.4 vs Group 2 = 6.5 ± 1.7, p < .05, Figure 4) and ϵ _pos peak_ and sec ϵ _pos peak_ were significantly decreased (Group 1 = 31.3 ± 4.2% vs Group 2 = 35.0 ± 7.6%, p < .01 and Group 1 = 18.4 ± 4% vs Group 2 = 21.4 ± 7.6%, p < .05) (Figure 5). ϵ _neg peak_ did not show significant differences (Group 1 = -12.9 ± 2% vs Group 2 = -13.6 ± 3%, p = ns). Interestingly, we found a significant correlation between ϵ _pos peak_ and age, but only in the control group (R = -.59, p < .001) and not in SSc patients (R = -.09, p = .57). Among echocardiographic parameters, no correlations were found between LA ϵ _pos peak_ and LV EF, nor E/e’, or LA indexed volume or PASP. No significant differences in LA ϵ _pos peak_ values were found between patients with and without Scl-70 antibodies (31.1 ± 4.2% vs 30.6 ± 4.1%, p = .75), nor between patients with limited and diffuse form (31.7 ± 3.9% vs 29.9 ± 5.7%, p = .79). No echocardiographic, STE or clinical parameter (including diffusing capacity for carbon monoxide) was significantly different between patients in NYHA class I and NYHA class II-III, nor between patients with normal and abnormal NT-proBNP values (only a trend in higher PASP was found in patients with abnormal NT-proBNP values: 18.7 ± 4.6 vs 25.3 ± 8.1 mmHg, p = .07).

**Table 1:** Clinical characteristics

**Clinical variables**	**Group 1 (SSc) 42 pts**	**Group 2 (Controls) 42 pts**	***p***
Age (years)	50 ± 14	49 ± 13	ns
Female gender (n,%)	40 (95%)	40 (95%)	ns
Systemic arterial hypertension (n,%)	10 (24%)	16 (38%)	ns
Diabetes (n,%)	0 (0%)	1 (2%)	ns
History of Smoking (n,%)	3 (7%)	6 (14%)	ns
Limited form n (%)	35 (83%)		
Diffuse form n (%)	7 (17%)		
Scl-70 antibodies n(%)	13 (31%)		
DLCO (%)	80.6 ± 23.2		
NTpro-BNP (pg/ml)	122 ± 135		

SSc: systemic sclerosis.

DLCO_ diffusing capacity of carbon monoxide.

NTpro-BNP- N-terminal B-type natriuretic peptide.

**Table 2:** Echocardiographic data

**Echo variables**	**Group 1 (SSc) 42 pts**	**Group 2 (Controls) 42 pts**	***p***
EF (%)	63.1 ± 4	66.1 ± 4	ns
LA indexed volumes (ml/m^2^)	24.9 ± 5.3	24.7 ± 4.4	ns
E/A	1.1 ± 0.4	1.3 ± 0.4	ns
E/e’	7.6 ± 2.4	6.5 ± 1.7	p˂0.05
PASP (mmHg)	24.1 ± 8	21 ± 7	ns
sec ϵ _pos peak_ (%)	18.4 ± 4	21.4 ± 7.6	p˂0.05
ϵ _neg peak_ (%)	-12.9 ± 2	-13.6 ± 3	ns
ϵ _pos peak_ (%)	31.3 ± 4.2	35 ± 7.6	p˂0.01

ϵ _pos peak:_ positive peak left atrial longitudinal strain; sec ϵ _pos peak:_ second positive peak left atrial longitudinal strain; ϵ _neg peak_ – negative peak left atrial longitudinal strain; A: mitral inflow late pulsatile Doppler wave; E: mitral inflow early pulsatile Doppler wave; e’: early diastolic mitral annular velocity; EF: ejection fraction; LA: left atrium; PASP: pulmonary artery systolic pressure; SSc: systemic sclerosis.

## Discussion

Our data show that SSc patients with normal left ventricular systolic function and without any significant cardiac abnormalities have reduced LA STE values, compared to a control group. STE may be a non-invasive, feasible method to assess early LA dysfunction in SSc patients. Such cardiac mechanic alterations are detectable in absence of changes in LA size.

### Pathophysiological mechanisms of LA dysfunction in SSc

The LA is a reservoir, a conduit and a pump, which plays an important role in modulating LV filling [22,23]. According to the Frank-Starling law, the LA pump function increases in the presence of mild LV diastolic dysfunction and then significantly decreases when LV diastolic dysfunction progresses to moderate or severe degrees [22-24]. Global LA function parameters, such as LA dimensions, area and volume, are good prognostic markers for predicting LV diastolic dysfunction [25,26]. However, an impairment of these parameters may appear late in the course of the disease. STE provides early, detailed information about LA mechanics. Several papers demonstrated the usefulness of STE in assessing LA function [10,27-30]. Inaba Y et al. showed that LA strain rate correlate with age, LA size and diastolic dysfunction in patients with atrial fibrillation [27]. LA strain rate was also an accurate predictor of recovery of LA contractility after cardioversion [28] and LA ϵ _pos peak_ was a significant predictor of postoperative atrial fibrillation in patients undergoing aortic valve replacement [29]**.**

In SSc, the myocardium is often characterized by patchy fibrosis, secondary to both repeated ischemia and/or immuno-inflammatory damage, inexorably leading to diastolic dysfunction. Episodes of ischemia in SSc are usually not due to epicardial coronary artery stenosis, but rather to microvascular dysfunction [31], since characteristic SSc vascular lesions result in major impairment of the microcirculation [4]. In addition to these fixed abnormalities, vasospasm of the small coronary arteries or arterioles may play a significant role in the early myocardial alterations in SSc [32]. Diastolic dysfunction is frequent in SSc [33-35], and is correlated with disease duration [36], whereas systolic dysfunction is present in only a minority of SSc patients [37]. Myocardial fibrosis in SSc has been reported in 50–80% of necropsy series [38-44], however the diagnosis of myocardial fibrosis can be challenging by non-invasive imaging. Up-to-now MRI is considered as the non-invasive gold standard imaging technique to assess myocardial fibrosis. Previous studies observed high percentages of myocardial fibrosis at MRI through detection of late gadolinium enhancement, although with different prevalence [38-44]. In a recent study, Ntusi et al. [45] found that although biventricular size and global function were preserved, there was impairment in the peak systolic circumferential and peak diastolic strain rate of the left ventricle, which inversely correlated with diffuse myocardial fibrosis indices at MRI.

### Clinical implications

Characterization of the LA has important prognostic implications [46,47]. LA enlargement is known to be associated with increased mortality in the general population [26]. Impaired LA function might be also an important predictor for the development of nonvalvular atrial fibrillation or other supraventricular arrhythmias [48], which are not infrequent in SSc patients. In our population, no patients had atrial fibrillation or other significant supraventricular arrhytmias. It would be interesting to see whether LA dysfunction can predict the development of supraventricular arrhythmias in this population.

In our study the LA reservoir (ϵ _pos peak_) and conduit (sec ϵ _pos peak_) function are impaired compared to controls. We may speculate that myocardial involvement and changes in the extracellular matrix may lead to early dysfunction of LA, even before the LA starts to dilate, since there was no difference between LA volume in the two groups. Early alternations of LA function can also mirror LV involvement in SSc. Myocardial fibrosis and consequent left ventricular diastolic dysfunction leads to the decrease of LA reservoir and conduit function and to the increase of LA pump function (Frank-Starling law).

The clinical meaning of an early assessment of LA mechanics in SSc by STE is still undetermined, and follow-up studies with a larger number of patients are needed to evaluate whether this finding has any prognostic implication. However, the use of a non-invasive, non-ionizing, and relatively simple method that lets us track LA function characteristics over time is intriguing. Speckle tracking allows a very sensitive estimation and monitoring of LA mechanics that may help us understand the progressive steps in the ongoing pathophysiological process leading to overt myocardial dysfunction. STE measurement has demonstrated to correlate also with the extent of LA fibrosis and remodeling [49]. This new echocardiographic analysis of LA function might also be employed as a biomarker in therapeutic trials, to assess efficacy in serial evaluations and follow-up of new therapeutic options.

### Comparison with previous studies

To the best of our knowledge, this is the first study evaluating LA function in SSc by STE strain analysis. Several reports had previously described subclinical LV and RV abnormalities in SSc patients, using TDI parameters and strain rate imaging [34,35,50-55]. Mele et al. showed that TDI-derived myocardial systolic deformation indices, based on strain and strain rate analysis and E/e’ ratio, are a valuable approach to detecting cardiac involvement in asymptomatic SSc patients [35]. D’Andrea et al. further confirmed that STE imaging can detect both RV and LV early myocardial involvement in SSc, as well as coronary flow reserve and brachial artery flow-mediated dilatation, as signs of early vascular impairment [53]. In another study, Shattke et al. employed isovolumetric acceleration, a novel tissue Doppler parameter, to detect early RV systolic impairment in SSc patients without pulmonary hypertension [51]. Kepez et al. found that SSc patients without pulmonary hypertension and overt clinical cardiac involvement had reduced myocardial strain and strain rate, despite normal 2D, conventional Doppler and TDI parameters [50]. Interestingly, Yiu et al. showed that subtle LV dysfunction, still assessed by STE strain analysis, is related to lower functional capacity and rhythm disturbances in patients with SSc [54]. Recently, Spethmann et al. showed that LV systolic impairment, as assessed by strain imaging, is primarily due to alterations in the basal LV segments in SSc patients with preserved LVEF [55].

All previous studies on speckle tracking in SSc patients never addressed LA function or dimensions, because they were focused on LV or RV function. It is true that assessment of both left and right ventricular dysfunction is the main target of cardiac assessment and represents the final result of all potential pathophysiological impairments. However, many of these alterations can be intercepted earlier by means of changes of the LA regional mechanical characteristics and function. A few previous studies had evaluated LA characteristics in SSc patients: Dimitroulas et al. showed that LA volume may be a useful noninvasive marker for the prediction of elevated pulmonary artery pressure in these patients [56]; impairment of electromechanical LA functions, including a prolonged intra-interatrial electromechanical delay and higher P-wave dispersion was also found in SSc patients, compared to a control group, confirming the increased risk of these patients for developing supraventricular arrhythmias [56].

### Study limitations

Some limitations of the present study should be highlighted. The study population was limited in size, since SSc is a relatively rare disease. Additionally, we excluded patients with signs of overt cardiac involvement, which could have affected strain and strain rate measurements. Therefore, our results cannot be extended to patients with heart failure or other concomitant cardiac disease. Finally, the study population was relatively old. It is well known that the occurrence of diastolic dysfunction increases with age. However, age was not different in the two groups, and interestingly, we found a significant correlation between ϵ _pos peak_ and age only in the control group and not in SSc patients. A main limitation is the lack of normal cut-off values for strain imaging. However, two studies report LA strain values in a healthy population, which can be considered as reference values [9,57,58]. Although very promising, a current significant limitation of strain imaging is inter-vendor variability; thus, up-to-date, strain analysis is still confined to the research field and has not yet been implemented in routine clinical practice.

## Conclusion

STE is a sensitive tool to assess impairment of LA mechanics, which is detectable in absence of changes in LA size and volume, and may represent an early sign of cardiac involvement in patients with SSc.

## Abbreviations

ϵ _pos peak_: Positive peak left atrial longitudinal strain; sec ϵ _pos peak_: Second positive peak left atrial longitudinal strain; ϵ _neg peak_: Negative peak left atrial longitudinal strain; A: Mitral inflow late pulsatile Doppler wave; BSA: Body surface area; E: Mitral inflow early pulsatile Doppler wave; e’: Early diastolic mitral annular velocity; EF: Ejection fraction; LA: Left atrium; LV: Left ventricle; NYHA: New York Heart Association; PASP: Pulmonary artery systolic pressure; SSc: Systemic sclerosis; STE: Two-dimensional speckle tracking echocardiography; TDI: Tissue Doppler imaging.

## Competing interests

The authors declare that they have no competing interests (there are not disclosures of any relationship with industry) relevant to the contents of this paper to disclose.

## Authors’ contributions

GA, LG, MHM, MC, LPB, AM, DM, SM, AMP, MMC, RS, EP, AV read and approved the final manuscript.
